# Want of Courtesy

**Published:** 1887-02

**Authors:** 


### WANT OF COURTESY.

Manners are not merely the surface politeness which distinguishes the man of the world or the woman of fashion. In fact, we might say that the two classes thus represented are, by their very nature, not calculated to inculcate true politeness, because one of its first requirements is sincerity—a feeling that is not expected to be found in the code of fashionable society.

The courtesy that gives the genuine tone to a person's manners is universally conceded to be prompted by a kind and sympathetic heart. The watchful consideration for the feelings of others must be felt itself before it can be sincerely expressed by kind acts and words. No vain, selfish, egotistical or cold-hearted man or woman can be truly polite. Tact, good sense, and good will are indispensable to the courtesy that charms, attracts, and opens the heart of its recipient.

As manners play so important a part in all conditions of life, and not only in society and the family circle, but in a man's professional and public career, it is greatly to be deplored that a want of courtesy is so apparent in the majority of the rising generation, and also in mature men and women, from whom one would reasonably expect better things, when we consider the position they occupy in life. It is one of the many evils—for an evil it is—that excites apprehension among the reformers of the period. It is apparent in the manner in which children, both juveniles and adults, at home and in public, address their parents. It is felt in the brusque and familiar tone with which the average young man greets the average young girls of his acquaintance. The deferential respect and chivalrous feelings that men should entertain towards all womanhood are too often conspicuously absent in the social intercourse of our young people.

The Bible strongly inculcates especial respect and courtesy to the aged, and pronounces a curse upon those who withhold from the hoary-headed the reverence to which their gray hairs give them a claim; but it is evident to all observing persons that there is a great decadence in that respectful feeling which should ever be manifested by youth to old age.

Professional men of all others, and especially medical men, should cultivate and endeavor to truly feel a frank and cordial courtesy towards all whom they come in contact, whether rich or poor, humble or exalted. No matter how high they stand in their respective professions, they are really the servants of the people; their prosperity largely depends upon the popular voice, and amid the revolutions of thought and sentiment now taking place in the world, the professional man who is thoroughly qualified not only to hold the position to which he aspires, but is regarded with general favor on account of his general and respectful manner towards all classes, is the man who is destined to win in the end. But apart from the utility of the question, it is most grateful to one's feelings to associate with persons whose charm of manner, expressive of a kind, noble heart, gives a nameless attraction to all they say or do and which beautifies even the sad and dreary way that many feet must tread through life. Courtesy is the flower of Politeness, and we cannot afford to trample its bloom and fragrance under our feet.

T. S. P.owell

---

*To the Members of the Medical Association of Georgia:*

Being appointed Chairman of the Section of Surgery of the Fifth District of the State Medical Association, which meets in Atlanta on the third Wednesday in April next, I desire to present as full a record of the past year's work as may be practicable.

I have to request those who may have met with surgical cases of interest to furnish me reports of the same, and, in the event complete details cannot be presented, notes of practical points will be acceptable.

Those who may be able to contribute surgical papers, giving their experience in the management of cases by general treatment or by operative means, will please to forward the titles of their articles without delay.

It is especially desirable to receive facts connected with abdominal and renal surgery from the practice of members; but notices of all cases involving any new principle of treatment, or exemplifying established modes of procedure are requested.

As the time is limited, immediate attention is desired. Address

J. McF. GASTON, M. D., Atlanta, Ga.

